# Horizontal Oxidation Diffusion Behavior of MEMS-Based Tungsten-Rhenium Thin Film Thermocouples

**DOI:** 10.3390/ma15145071

**Published:** 2022-07-21

**Authors:** Yong Ruan, Meixia Xue, Jiao Teng, Yu Wu, Meng Shi

**Affiliations:** 1 Department of Precision Instruments, Tsinghua University, Beijing 100084, China; 18811346921@163.com (Y.W.); shimqwe@163.com (M.S.); 2 Department of Materials Physics and Chemistry, University of Science and Technology Beijing, Beijing 100083, China; g20208471@xs.ustb.edu.cn (M.X.); tengjiao@mater.ustb.edu.cn (J.T.)

**Keywords:** thin film thermocouples, microelectromechanical system, horizontal oxidation

## Abstract

Tungsten-rhenium thin film thermocouples (TFTCs) are well suited for the surface temperature monitoring of hot components due to their small size, rapid response and low cost. In this study, a tungsten-rhenium TFTC with SiC protective film on all parts except the pads was fabricated by a microelectromechanical system (MEMS) process. During the low to medium temperature (−40 °C to 500 °C) repeatability test phase, the thermal voltage from the TFTC agreed well with that of the standard tungsten-rhenium thermocouple. However, during the high temperature test phase, the TFTC lost electronic response at around 620 °C. Failure analysis of the TFTC tested at 620 °C was performed by microscopy, scanning electron microscope (SEM), energy dispersive spectroscopy (EDS), laser scanning confocal microscope (LSCM) and statistics. The results showed that the pads were oxidized without the protective layer, the number of oxidized protrusions distributed in this TFTC from the pad to the node decreases more and more slowly and the size of the oxidized protrusions also becomes smaller and smaller. This demonstrates the presence of horizontal oxidation diffusion in TFTCs, further illustrating the importance of pad protection and provides a direction for the subsequent structural optimization and the extension of the service life of TFTCs and other sensors.

## 1. Introduction

High-temperature monitoring in confined spaces, such as a combustion chamber of an engine, applies harsh requirements on thermosensitive parts [1,2,3,4,5]. Compared to conventional wire and foil thermocouples, thin film thermocouple (TFTC) by microelectromechanical system (MEMS) process has the advantages of fast response speed and small size due to its low thermal mass and process [6,7,8,9]. Moreover, the thin films deposited by the MEMS process are exactly uniform without the problem of surface particles, which is more conducive to the production of large areas of films [10]. TFTCs were first proposed by Hackenann, a German researcher, during World War II to measure the temperature changes in the chamber wall of a gun [11]. A platinum–rhodium TFTC was fabricated on the alumina substrate by micromachining technology [12], which could operate at the temperature up to 1300 °C, but the high material cost limited its application to a certain extent. Tian Bian et al. fabricated a tungsten-rhenium TFTC-based SiC by magnetron sputtering, which had a potential output of 9 mV at 900 K with a protective tube [13]. The protective film on the surface of tungsten-rhenium TFTCs was proposed to effectively prevent the oxidation and evaporation of tungsten-rhenium TFTCs at high temperatures [14]. An In_2_O_3_-In_2_O_5_Sn (ITO) TFTC was fabricated with a high Seebeck coefficient of 44.5 μV/°C [15], but with a low repeatability.

Consequently, compared to In_2_O_3_-ITO thermocouples, tungsten-rhenium materials have been maturely used in linear thermocouples and have high repeatability. Compared to platinum-rhodium thermocouples, tungsten-rhenium thermocouples have higher sensitivity and greatly reduce costs. However, more research is needed to determine its high temperature oxidation characteristics. In this study, we fabricated the TFTCs with SiC as the substrate, W-5Re (95 wt% tungsten-5 wt% rhenium) and W-26Re (74 wt% tungsten-26 wt% rhenium) as the sensitive layer and SiC as the protective layer by a microelectromechanical system (MEMS) process. We evaluated the process, tested the thermoelectric effect of TFTCs and performed a failure analysis. The horizontal oxidation phenomenon was also analyzed by optical microscope, scanning electron microscope (SEM), energy dispersive spectrometer (EDS) and laser scanning confocal microscope (LSCM). It is concluded that it is important to add a thick protective layer on the pads and thermocouple lines.

## 2. Materials and Methods

The W-5Re and W-26Re sensitive film had a thickness of 0.3 μm and a wire width of 400 µm. The sizes of the junction and pads were 100 μm × 400 μm and 1200 μm × 1200 μm, respectively. The SiC protective film had a thickness of 0.1 μm. The size of the TFTC lines was 5.5 mm × 15.5 mm.

### 2.1. Fabrication of TFTCs

**Figure 1:**
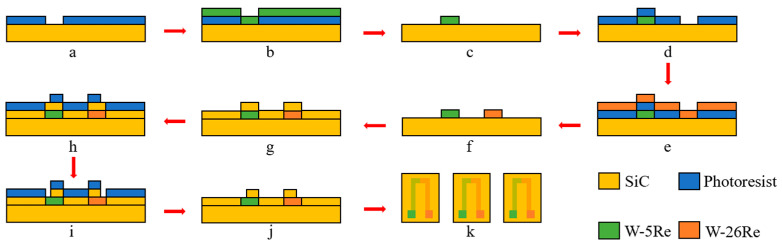
Fabrication process of tungsten-rhenium TFTCs (**a**–**k**).

The tungsten-rhenium TFTCs were prepared based on the MEMS process. The flowchart of the fabrication process was shown in Figure 1 and described as follows. The SiC substrate had a diameter of 6 in and a thickness of 0.4 mm, which was cleaned ultrasonically in the bath of pure alcohol and then acetone, respectively. The relevant parameters [16,17] are shown in Table 1.

(a) First lithography process: The positive photoresist (PR) of AZ 5214 with a thickness of 2.8 μm was spin-coated and soft baked on the SiC substrate. Then, it was patterned using ultraviolet (UV) lithography and developing processes. The positive patterns of TFTCs were obtained.

(b) A magnetron deposition system (DENTON Explorer-14, America) was used to deposit films in this paper. The W-5Re film with a thickness of 0.3 μm was deposited on the above substrate by DC magnetron sputtering.

(c) The remaining PR was removed using acetone solution.

(d) Second lithography process: The AZ 5214 with a thickness of 2.8 μm was spin-coated and soft baked again. It was patterned using UV lithography and developing processes. Then, the negative patterns of TFTCs were obtained.

(e) The W-26 Re film with a thickness of 0.3 μm was deposited on the substrate by DC magnetron sputtering.

(f) The remaining PR was removed using acetone solution again. Additionally, the substrate was annealed at 300 °C for two hours under N_2_.

(g) The SiC film with a thickness of 0.1 μm was deposited on the substrate by RF magnetron sputtering.

(h) Third lithography process: The AZ 5214 with a thickness of 2.8 μm was spin-coated and soft baked once again. It was patterned by UV lithography and developing processes. Then, the pad patterns of TFTCs were obtained.

(i) The cold terminal pads of TFTCs were exposed by etching 0.1 μm with reactive ion etching machine.

(j) The remaining PR was once again removed with acetone solution.

(k) The wafers were cut on the extended scribe path to obtain many individual TFTCs.

### 2.2. Compensation Wire Connection and Initial Package

**Figure 2:**
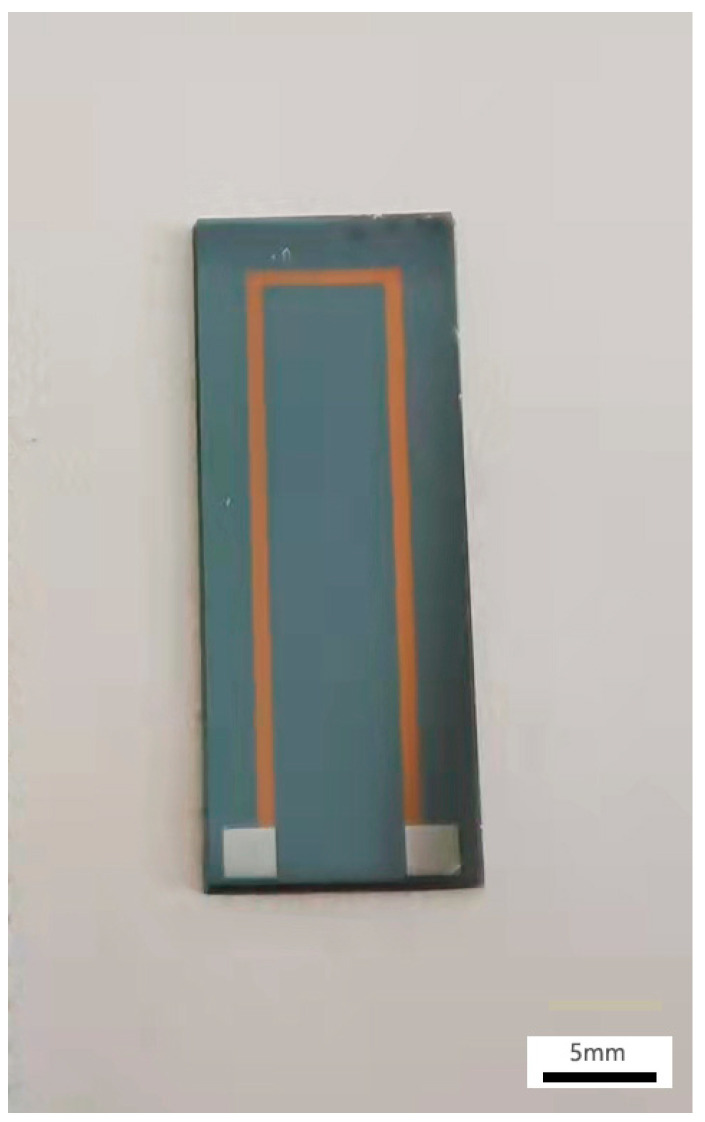
The fabricated TFTC by MEMS process.

**Figure 3:**
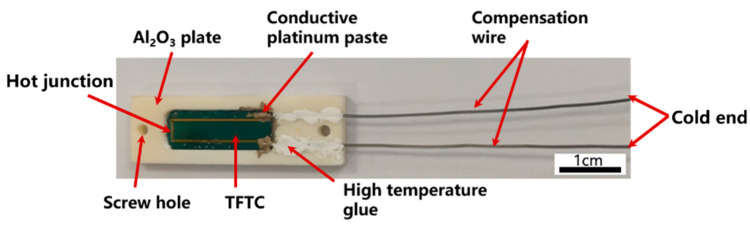
Connection of compensation wires.

The fabricated TFTC by MEMS process is showed in Figure 2, which was put into a customized Al_2_O_3_ plate for lead connection as shown in Figure 3. The Al_2_O_3_ plate was designed with recesses to allow the TFTC and homogeneous wires as compensation wires to fit exactly into them, and screw holes were reserved for subsequent packaging. The TFTC and leads were bonded to the ceramic plate using high-temperature glue to improve the mechanical strength of the overall sensor connection. The platinum paste was applied to provide electrical connections between the pads and compensation wires, and processed excellent electrical conductivity even at 1000 °C. After completing the above process, we covered it with matching Al_2_O_3_ covers with the same pre-drilled screw holes and screwed them together with ceramic screws, which completed the initial sandwich structure of the TFTC package.

The packaged TFTC was placed in a quartz tube and tested at thermostats, while there were standard thermocouples to control the temperature of these thermostats in real time.

## 3. Results and Discussion

### 3.1. Characterization of TFTC Films by MEMS

**Figure 4:**
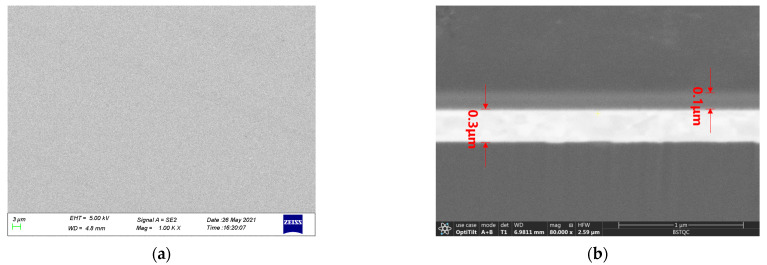
SEM micrographs of TFTC by MEMS. (**a**) Surface; (**b**) Cross section.

As shown in Figure 4a, the surface of the TFTC film is flat and smooth without obvious cracks and stray particles, which is uniformly covered on the SiC substrate [18]. From Figure 4b, the thickness of the tungsten-rhenium film is about 0.3 μm, and the protective layer SiC is about 0.1 μm. The film is dense without loose voids, while the layers are clearly distinguished without diffusing from each other, indicating that the above process design is very suitable for TFTC.

### 3.2. Thermoelectric Testing

**Figure 5:**
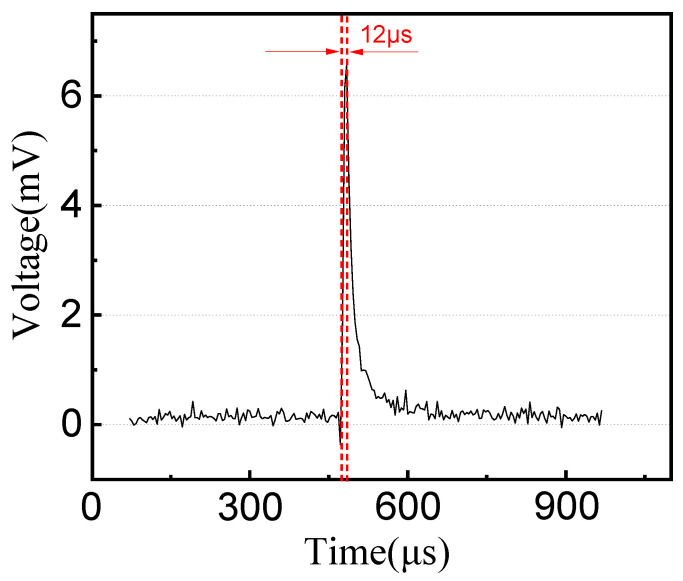
The response time of the TFTCs.

**Figure 6:**
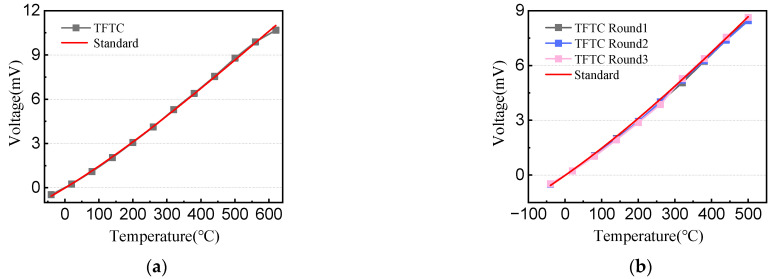
Comparison of voltage–temperature curves of TFTC and standard wire thermocouples. (**a**) −40~620 °C; (**b**) −40~500 °C.

The working principle of the TFTC is the Seebeck effect (thermoelectric effect) theory [19]. Two conductors (or semiconductors) of different materials form a closed loop. When the temperature of the cold end and the hot junction are different, a thermoelectric potential will be generated in the loop, and then the temperature information is indirectly reflected by measuring the voltage difference in the loop. As shown in Figure 5, the response time of the TFTC is 12 μs, which is 12 times faster than the response time of the same type of thermocouple made by Zhang Zhongkai et al. [20]. The voltage–temperature curves of the TFTC and the standard wire thermocouples are shown in Figure 6 and the sensitivity is 17.19 μV/°C. As shown in Figure 6a, the voltage–temperature curves of the TFTC and standard wire thermocouple have the same trend, they overlap each other and almost appear to be a single line, which indicates that the fabricated TFTC has a high accuracy [21].

The thermoelectric testing starts at −40 °C and takes about 3 h. When the temperature reaches 620 °C, the TFTC shows voltage instability and finally loses the electrical signal. Although there is no electrical signal between the two pads, testing this TFTC with a multimeter reveals that the platinum paste to the end of the compensation wires is still conductive. Therefore, this is a failure of the sensor itself.

In order to verify whether the failure of TFTC is related to high temperature or long use time, we repeatedly test it at 500 °C to ensure it works properly. As shown in Figure 6b, TFTC continues to work after three cycles, about 9 h, and the accuracy remains high, indicating that its failure is mainly related to high temperature.

### 3.3. Failure Analysis

#### 3.3.1. Optical Observation

**Figure 7:**
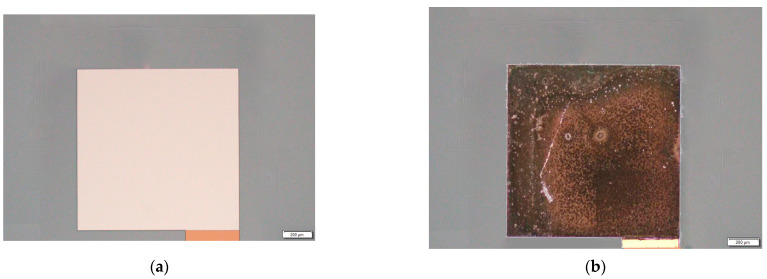
Optical images of TFTC pads. (**a**) Before 620 °C; (**b**) After 620 °C.

Changes in the fine structure of the TFTC are no longer easily observable after the compensation wire is connected. To facilitate the observation, TFTC molds subjected to the same test environment are used in the subsequent analysis and inspection. The optical images of TFTC pads before and after 620 °C are shown in Figure 7.

After testing, the surface of the pads becomes burnt black from the yellow before testing, and the dense film on the surface of the pads becomes a layer of powder-like particles. The resistance of TFTC before the test is 178 Ω, but exceeds the range of the test instrument (20 MΩ) after the test. Analysis suggests the lack of a protective layer on the pad due to the oxidation of the tungsten-rhenium metal film to a black powder in the air at about 620 °C, resulting in no electrical response.

**Figure 8:**
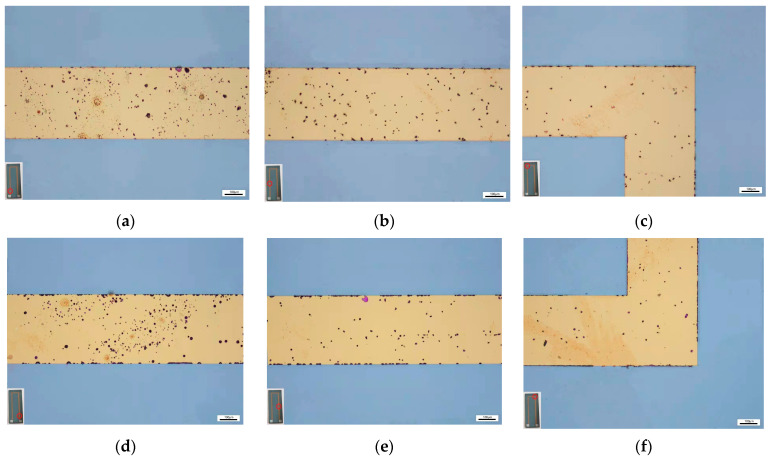
Optical images of the TFTC line. (**a**) The bottom of the TFTC left line; (**b**) The middle of the TFTC left line; (**c**) The top of the TFTC left line; (**d**) The bottom of the TFTC right line; (**e**) The middle of the TFTC right line; (**f**) The top of the TFTC right line.

**Figure 9:**
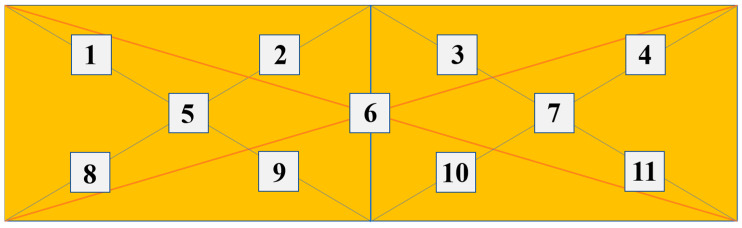
Model diagram of the sampling method.

Figure 8 shows the optical images of TFTC left and right lines at different positions. The bottom, middle and top images of each side line are equally spaced samples, spaced 500 μm apart. There are plenty of black spots appearing on the lines of TFTC. Since the distribution of black spots is not particularly uniform, the paper adopts the sampling model shown in Figure 9 to calculate the quantity in order to make the results more accurate. For each image, 11 rectangular bits of 70 μm × 70 μm are selected as sampling points (images in Figure 8c,f are straightened during sampling).

**Figure 10:**
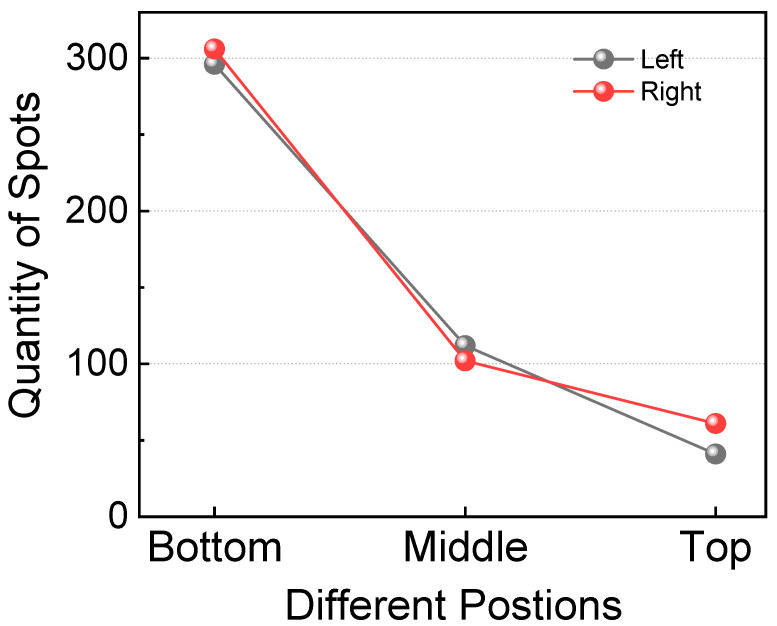
The relationship between the quantity and position of black spots.

The quantity results of black spots at different sampling points are shown in Table 2. As shown in Figure 10, the quantity of black spots in each graph of Figure 8 is obtained after the average calculation, which gradually decreases from the bottom (near the pad) to the top (near the node), and decreases faster from the bottom to the middle than from the middle to the top. Therefore, it is speculated that the TFTC not only undergoes vertical oxidation through the protective layer, but also horizontal oxidation from the pad to the node direction. Additionally, the closer this oxidation is to the pad, the faster it oxidizes.

#### 3.3.2. EDS and SEM Analysis

**Figure 11:**
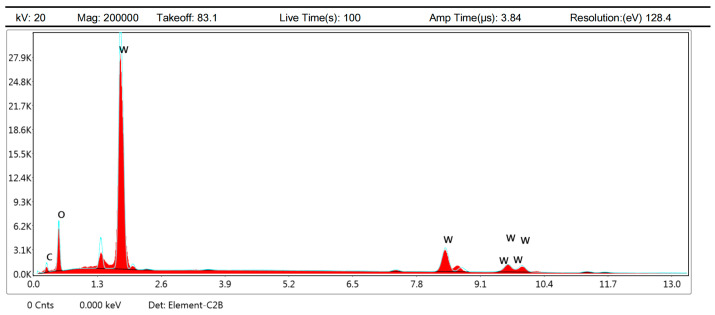
The EDS pattern of spots.

After EDS analysis, as shown in Figure 11, it is found that the main components of these spots are O and W elements, indicating that they are mainly oxidation products of W and Re elements, which causes the color change. The reason that the Re element is not detected is that the content of the Re element is originally less than that of the W element, and the rhenium oxide formed by the reaction at high temperature volatilizes due to the low sublimation point, which is consistent with the perspective of Dai Min [22].

**Figure 12:**
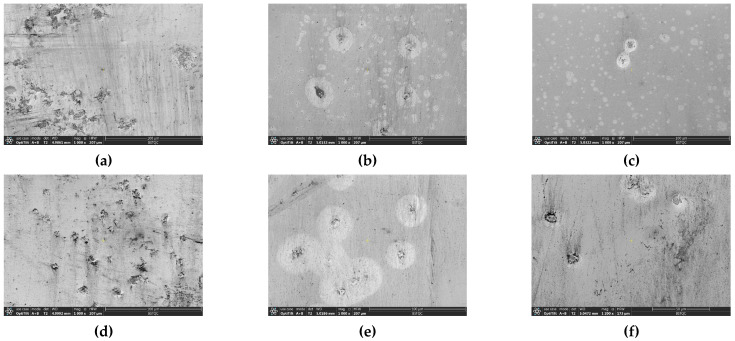
SEM images of the TFTC line. (**a**) The bottom of the TFTC left line; (**b**) The middle of the TFTC left line; (**c**) The top of the TFTC left line; (**d**) The bottom of the TFTC right line; (**e**) The middle of the TFTC right line; (**f**) The top of the TFTC right line.

**Figure 13:**
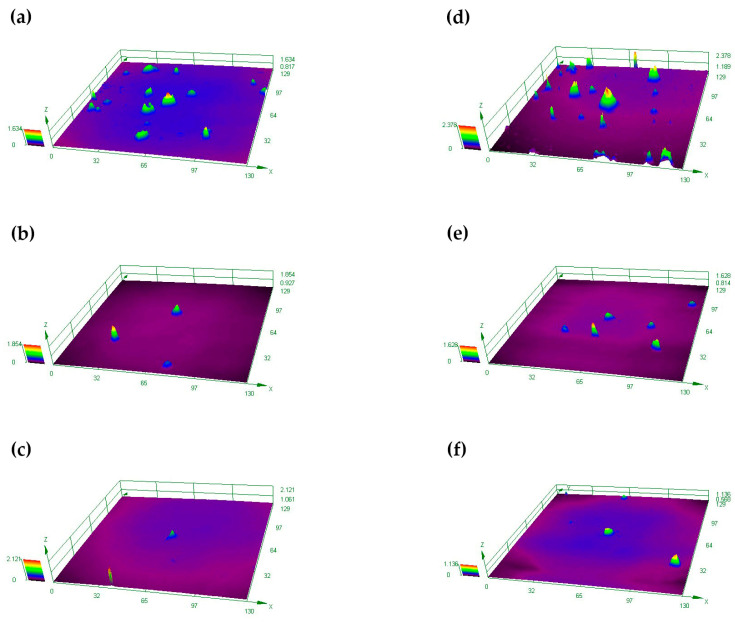
LSCM images of the TFTC line. (**a**) The bottom of the TFTC left line; (**b**) The middle of the TFTC left line; (**c**) The top of the TFTC left line; (**d**) The bottom of the TFTC right line; (**e**) The middle of the TFTC right line; (**f**) The top of the TFTC right line.

**Figure 14:**
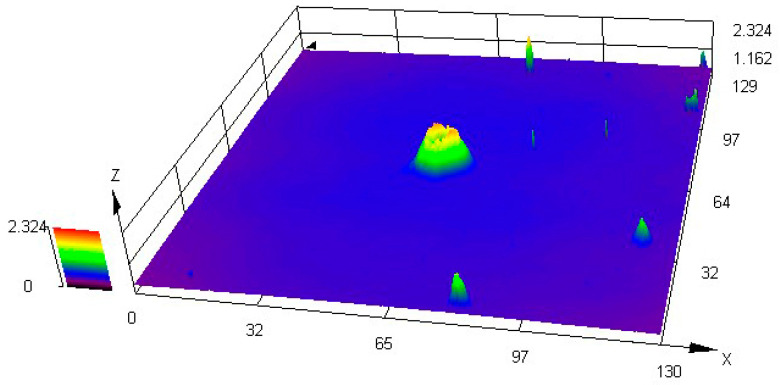
LSCM images of the large spot.

As shown in Figure 12 and Figure 13, it is observed by SEM and LSCM that the black spots are oxide protrusions whose number at the bottom position is higher and the size is larger, while the number of protrusions at the top is relatively less and the size is smaller. The oxide in some of the large-sized protrusions has fallen off, forming pits with crater morphologies in Figure 14, which also demonstrates the formation process of small-sized oxide protrusions, middle-sized protrusions and large pits with crater morphologies. The decreasing number and size of protrusions from the bottom to the top indicate that the oxidation of TFTC from the bottom to the top is getting lighter. Thus, not only does vertical oxidation occur through the protective layer [23], but horizontal oxidation also occurs from the bottom to the top direction.

Therefore, we found that the protective layer of the TFTC needs to be thickened to resist oxidation at high temperatures [20], and the pads also need to be protected because not only does the pad undergo oxidation, but the TFTC also suffers from horizontal oxidation. This finding also applies to other sensors.

## 4. Conclusions

A tungsten-rhenium TFTC with a SiC protective film on all parts except the pads was fabricated by the MEMS process, and initial package and testing were performed. The Seebeck coefficient was 17.19 μV/°C and the response time was up to 12 μs. Additionally, the repeatability was still consistent with the standard curve under multiple cycle tests. However, during the high temperature test phase, the TFTC failed at around 620 °C. In this study, failure analysis is performed and it is verified that horizontal oxidation diffusion in the TFTC occurs, illustrating the importance of pad protection and providing a direction for the subsequent structural optimization and the extension of the service life of TFTCs and other sensors.

## Figures and Tables

**Table 1:** Sputtering parameters.

	Target-Base Distance (mm)	Background Pressure (Torr)	Working Pressure (Torr)	Sputtering Power (W)	Ar2 Flow Rate (sccm)
W-5Re	60	2.8 × 10^−7^	5 × 10^−3^	250	45
W-26Re				200	
SiC				200	

**Table 2:** The quantity of black spots at different sampling points.

Serial Number	Quantity of Spots										
	1	2	3	4	5	6	7	8	9	10	11
a	6	2	1	4	2	4	2	1	1	6	2
b	0	0	0	2	3	1	2	0	1	2	2
c	0	1	1	1	0	0	0	1	0	0	2
d	0	2	6	2	3	4	1	2	4	6	0
e	2	2	0	0	4	2	0	0	0	0	1
f	1	0	1	1	1	0	1	1	0	0	0
